# Noninvasive Preimplantation Genetic Testing for Aneuploidy (niPGT-A): The first Brazilian baby

**DOI:** 10.5935/1518-0557.20200074

**Published:** 2020

**Authors:** José Gonçalves Franco Jr., Laura Diniz Vagnini, Claudia Guilhermino Petersen, Adriana Renzi, Maria C.T. Canas, Bruna Petersen, Juliana Ricci, Andreia Nicoletti, Camila Zamara, Felipe Dieamant, João Batista Alcantara Oliveira

**Affiliations:** 1 Center for Human Reproduction - Prof. Franco Jr., Ribeirão Preto, Brazil.; 2 Paulista Center for Diagnosis, Research, and Training, Ribeirão Preto, Brazil.

**Keywords:** Noninvasive Preimplantation Genetic Testing for Aneuploidy, niPGT-A, PGT-A

## Abstract

Recently, a new technology known as the Noninvasive Preimplantation Genetic Testing for Aneuploidy (niPGT-A) emerged, using cell-free DNA present in the spent culture media of human blastocysts. Unlike PGT-A, in which only trophectoderm cells are used, niPGT-A reflects the ploidy state of these cells and internal cell mass, suggesting that this new technology may be less prone to error, being more reliable than the invasive test. The aim of the present study was to report the first occurrence of childbirth following niPGT-A in Brazil.

## INTRODUCTION

After several years of using Preimplantation Genetic Testing for Aneuploidy (PGT-A), many concerns remain, such as risks due to invasive action and difficulties in the correct interpretation of mosaicism, which may lead to errors in the analysis of false-positive and -negative results (Greco *et al*., 2015; Franco, 2015; 2019; Kang *et al*., 2016; Gleicher *et al*., 2015; 2016; 2018; Paulson, 2017; Rosenwaks *et al*., 2018; Munné *et al*., 2017; 2019). Recently, a new technology known as the Noninvasive Preimplantation Genetic Testing for Aneuploidy (niPGT-A) emerged, using cell-free DNA present in the spent culture media of human blastocysts (Fang *et al*., 2019; Farra *et al*., 2018; Feichtinger *et al*., 2017; Franco, 2019; Franco *et al.*, 2020; Ho *et al*., 2018; Huang *et al*., 2017; Olcha *et al*., 2020; Shamonki *et al*., 2016; Vagnini *et al.*, 2020; Xu *et al*., 2016). Unlike PGT-A, in which only trophectoderm cells are used, niPGT-A reflects the ploidy state of these cells and internal cell mass, suggesting that this new technology may be less prone to error, being more reliable than the invasive test.

The aim of the present study was to report the first occurrence of childbirth following niPGT-A in Brazil.

## CASE REPORT

### Patients

A couple (J.S., a 37-year-old woman and B.S., her 36-year-old husband) was admitted in 2019 for an initial medical consultation. They had an 8-year history of secondary infertility due to the tuboperitoneal factor and endometriosis.

-She presented a history of ectopic pregnancy, which was treated by left salpingoopherectomy.

Tests:

Antral follicle count: 11; Serum Anti-Müllerian hormone concentration: 1.44 ng/mL; Body mass index (BMI): 26.84 kg/m2.

Laparoscopy: normal uterus, lysis of adhesions, removal of the remainder of the left tube, sites of disseminated endometriosis in the pelvis. Right tube underwent previous Hysteroscopy: Normal uterine cavity.

Hormonal Measurement: TSH = 1.34 µIU/mL; Free T4 = 0.71 ng/dL; Prolactin = 20.7 ng/mL.

Karyotype: 46,XX

-He did not present relevant data in his clinical history.

Tests:

Spermogram: concentration: 21×106/mL, motility: 68% progressive, morphology (by MSOME): Normal forms: 0; forms with vacuoles occupying >50 of the nuclear area: 46%.

The sperm DNA fragmentation index: 11%.

Protamination index: 28% (low).

Karyotype: 46,XY.

### IVF Treatment

The patient underwent controlled ovarian stimulation following the long GnRH agonist (Lupron^®^, Abbott Laboratories, Brazil) protocol and using recombinant FSH (GonalF^®^, Merck Serono, Italy) and recombinant FSH+LH (Pergoveris^®^, Merck Serono, Italy) for 08 days. When the dominant follicles reached 18 mm, final oocyte maturation was triggered using a single dose of recombinant hCG (rhCG) 250 µg (Ovidrel^®^, Merck Serono, Italy).

Oocyte pick-up was conducted 36h post-hCG and resulted in the collection of 11 cumulus-oocyte complexes. On the date of aspiration, the seminal sample presented 65% of progressive motility and a concentration of 35×106/mL and 61% of progressive motility. After one hour of retrieval, all oocytes were denudated using hyaluronidase 40IU, for 2-3 minutes. The corona cells were completely removed using a 150µm stripper to minimize patient contamination. Eleven MII oocytes were identified and inseminated via intracytoplasmic morphologically selected sperm injection (IMSI), as described previously (Oliveira *et al*., 2011). At 18h post-ICSI, the morphological assessment indicated 8 fertilized oocytes presenting two pronuclei and the extrusion of the second polar body. On day 2, the two best-scoring embryos were transferred.

The exceeding embryos were cultured until reaching blastocyst stage, at 37ºC in an atmosphere containing 7% CO2 and 5% O2. A total of 3 embryos arrived at blastocyst stage and were cryopreserved. Two of them were submitted to cryopreservation after their respective spent culture medium was collected for genetic analysis (niPGT-A).

After 14 days of transfer, the serum ß-hCG level was negative. On account of the negative result, we decided to transfer the frozen embryos analyzed by niPGT-A.

### Noninvasive preimplantation genetic testing for Aneuploidy (niPGT-A)

#### IVF Laboratory

A total of 5 exceeding embryos were cultured from day 3 to 5. On Day 3, the cleaved embryos were re-evaluated for complete cumulus cell removal by washing them individually three times to remove any remaining attached granulosa cells surrounding the embryos. Afterward, the embryos were transferred to individual wells containing 20 µL of fresh medium in GPS^®^ dishware (SP38-010) under oil and cultured until they reached blastocyst stage on the 5th day.

#### Sample collection

 All spent medium from the blastocysts, left in each GPS^®^ dish microdroplet, was loaded into a previously identified sterile 0.2 mL PCR tube containing 5 µL of lysis buffer using individually stretched pipettes. The PCR tubes were stored at -20ºC for at least 24hrs before shipping to the laboratory for genetic evaluation. A sample (20 µL) of the medium from one individual well (GPS^®^ dish), in which no embryos were cultured, was also collected and used as a control. During medium collection, all the procedures were handled under sterile conditions in a laminar flow cabinet using a mask, cap, gloves, and sterile materials.

##### Whole Genome Amplification and DNA sequencing

The free DNA secreted into the culture medium by each blastocyst was amplified using the NICS Sample Preparation Kit (Yikon Genomics) based on MALBAC technology. After whole genome amplification, the DNA was measured using a Qubit 2.0 fluorometer (Thermo Fisher Scientific) and subjected to Next-Generation Sequencing (NGS) using the Illumina MiSeq^®^ platform.

##### Data analysis

 The ChromGo (Yikon Genomics, China) software was used to analyze sequencing data and report chromosomal abnormalities. This software allows the evaluation of entire chromosomes, analysis of each chromosome’s short and long arms, and the detection of deletion or duplication >10Mb, in addition to enabling the determination of embryo sex and the presence of mosaicism.

##### niPGT-A results (Figure 1):

**Figure 1:**
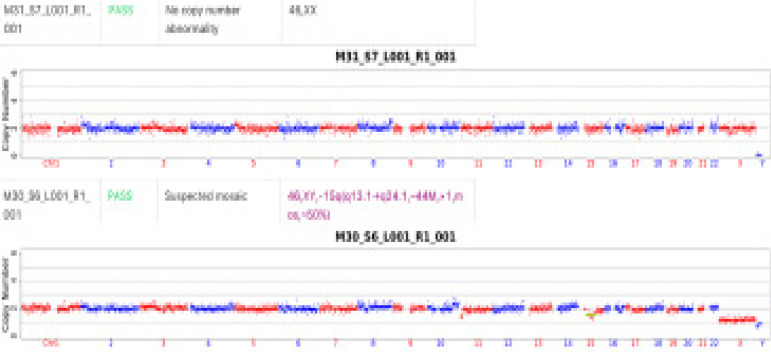
niPGT-A results

Blastocyst 1(M31): 46,XX

Blastocyst 2(M30): 46,XY,-15q(q13.1→q24.1,~44M,×1,

mos,~50%). Mosaic partial monosomy or deletion chromosome 15 in ≈50% of cells.

##### Frozen Embryo transfer

The endometrium was prepared through the daily oral administration of 6 mg of estradiol valerate (Cicloprimogyna^®^, Schering, São Paulo, Brazil) starting on the first day of the menstrual period. On the 14^th^ day of use, transvaginal ultrasonography showed an endometrium thickness of 7.6 mm and a triple-line pattern. From then on, the patient began daily intravaginal administration of progesterone (Utrogestan^®^, Besins Healthcare, Brazil). On the 5^th^ day of progesterone administration, the two blastocysts diagnosed as 46,XX and 46,XY Mosaic by niPGT-A were warmed up and transferred. After 14 days, beta-hCG quantification was measured at 427.75 mIU/mL.

##### Pregnancy Evolution

-USG 6 weeks of evolution: Topic 6-week twin pregnancy. Embryo I, fetal heartbeat present. Embryo II, fetal heartbeat present.

-USG 7 weeks: arrested development of one of the embryos. 

-12 weeks:

USG: Nuchal translucency 1.65 mm.

Biochemical Evaluation for trisomies: Low risk.

Fetal sexing: female sex.

-08/04/2020:39 weeks: birth of a healthy girl with 3.540kg and 50 cm. Apgar index 1^st^ min: 9/ 5^th^ minute - Clinical evaluation of the newborn: no alterations.

##### Comments

Recently, Xu *et al*. (2016) described the possibility of non-invasive chromosomal screening (niPGT-A) by obtaining and sequencing free DNA released by the embryos into the culture medium (without the need for embryo biopsy), creating a new, non-aggressive and elegant perspective for pre-implantation genetic diagnosis. This case description adds to several recently published articles that describe the birth of healthy children from euploid blastocysts selected by niPGT-A in ICSI programs, and also for couples with genetic alterations (Xu *et al*., 2016; Fang *et al*., 2019). niPGT-A, which only involves collecting embryo culture medium, can be, at least potentially, used in all ICSI cycles.

An underlying issue is the possibility of niPGT-A overcoming problems inherent in classical PGT-A. The need for biopsies, the potential damage to the trophectoderm, and the implantation process, in addition to requiring embryologists with considerable training and experience to perform embryo manipulation, are among the issues regarding PGT-A. On the other hand, queries remain concerning the future risks of invasive action of cells removed during the biopsy. In animals, some data suggest that embryonic biopsies may be associated with changes in the fetal neural tube or adrenal development (Wu *et al*., 2014; Zeng *et al*., 2013). Also, PGT-A determines the embryonic genetic state by analyzing some trophectoderm cells, which, in fact, do not originate the embryo, but become part of supporting structures, such as the placenta and membranes. Undoubtedly, it would be difficult to accurately assess the presence of mosaicism generating significant levels of false-positive results and, thus, causing a real possibility of discarding healthy embryos.

Current knowledge regarding the source of free DNA in embryonic culture media is limited. However, we cannot deny that niPGT-A can detect genomic DNA, not only from trophectoderm cells but also from internal cell mass (ICM) of the blastocyst. Thus, niPGT-A could provide more accurate information on embryonic ploidy. Nonetheless, the precise contribution of each of these groups of cells to the final concentration of free DNA is challenging to assess.

Apoptosis, at least in theory, is implicated as the primary source of free DNA collected for the execution of niPGT-A. The percentage and speed in eliminating abnormal cells (self-correction) can be associated with the ploidy characteristics of the embryos. Bolton *et al*. (2016) showed in a mosaic mouse model that aneuploid and euploid cells from the ICM, as well as from the trophectoderm, undergo apoptosis. These researchers also showed that a larger percentage of ICM cells became apoptotic when compared to trophectoderm cells, regardless of whether they were aneuploid (41.4% vs. 3.3%, respectively) or euploid (19.5% vs. 0.6%, respectively).

Some studies have also reported better results with niPGT-A. Huang *et al*. (2019), using embryos sent for research, compared the results of niPGT-A and PGT-A. False-positive results were significantly less common in niPGT-A (20%) than in PGT-A (50%) when both were compared with the total blastocyst screening (control). Meanwhile, a 100% agreement was observed between the niPGT-A and the genetic analysis of the donated blastocyst regarding euploid embryo diagnosis.

Another multicentric study (Vagnini *et al*., 2020) found a similar result. This cohort study included a total of 37 blastocysts, vitrified on day 5, which were previously biopsied for PGT-A and presented a diagnosis of aneuploidy. The ploidy status results obtained for the culture medium and whole embryo were compared in order to determine the accuracy of niPGT-A for screening chromosomal abnormalities in each embryo. When comparing the niPGT-A and whole embryo sequencing results, the positive predictive value (PPV) was 93.5%, and the FPR was 6.5%. On the other hand, comparing the whole embryo and PGT-A results, the PPV was 78.4%, and the FPR was 21.6%. Both niPGT-A and PGT-A had a negative predictive value (NPV) of 100% and a false-negative rate (FNR) of 0%. Thus, when DNA sequencing from whole embryo cells was used as the gold-standard, the FPR of niPGT-A was 3.32-times smaller than that obtained with PGT-A.

niPGT-A is a method that avoids trophectoderm biopsy trauma and seems to provide more accurate results regarding the ploidy status of the whole embryo. Therefore, niPGT-A should be considered as the test of choice for the genetic selection of the embryo.
